# Critical low temperature for the survival of *Aedes aegypti* in Taiwan

**DOI:** 10.1186/s13071-017-2606-6

**Published:** 2018-01-08

**Authors:** Pui-Jen Tsai, Tang-Huang Lin, Hwa-Jen Teng, Hsi-Chyi Yeh

**Affiliations:** 1 Center for General Education, Aletheia University, New Taipei City, 25103 Taiwan, Republic of China; 2 Center for Space and Remote Sensing Research, National Central University, Jhongli, 32001 Taiwan, Republic of China; 3 Center for Diagnostics and Vaccine Development, Centers for Disease Control, Taipei, Taiwan, Republic of China

**Keywords:** *Aedes aegypti*, Moderate resolution imaging spectroradiometer, Inverse distance weighting, Local polynomial interpolation, Radial basis function, Ordinary kriging, Phi coefficient

## Abstract

**Background:**

Taiwan is geographically located in a region that spans both tropical and subtropical climates (22–25°N and 120–122°E). The Taiwan Centers for Disease Control have found that the ecological habitat of *Aedes aegypti* appears only south of 23.5°N. Low temperatures may contribute to this particular habitat distribution of *Ae. aegypti* under the influence of the East Asian winter monsoon. However, the threshold condition related to critically low temperatures remains unclear because of the lack of large-scale spatial studies. This topic warrants further study, particularly through national entomological surveillance and satellite-derived land surface temperature (LST) data.

**Methods:**

We hypothesized that the distribution of *Ae. aegypti* is highly correlated with the threshold nighttime LST and that a critical low LST limits the survival of *Ae. aegypti*. A mosquito dataset collected from the Taiwan Centers for Disease Control was utilized in conjunction with image data obtained from the moderate resolution imaging spectroradiometer (MODIS) during 2009–2011. Spatial interpolation and phi coefficient methods were used to analyze the correlation between the distributions of immature forms of *Ae. aegypti* and threshold LST, which was predicted from MODIS calculations for 348 townships in Taiwan.

**Results:**

According to the evaluation of the correlation between estimated nighttime temperatures and the occurrence of *Ae. aegypti*, winter had the highest peak phi coefficient, and the corresponding estimated threshold temperatures ranged from 13.7 to 14 °C in the ordinary kriging model, which was the optimal interpolation model in terms of the root mean square error. The mean threshold temperature was determined to be 13.8 °C, which is a critical temperature to limit the occurrence of *Ae. aegypti*.

**Conclusions:**

An LST of 13.8 °C was found to be the critical temperature for *Ae. aegypti* larvae, which results in the near disappearance of *Ae. aegypti* during winter in the subtropical regions of Taiwan under the influence of the prevailing East Asian winter monsoon.

## Background

The mosquito *Aedes aegypti* (L.) is the primary urban vector of several globally critical arboviruses, including dengue virus (DENV) and the yellow fever virus, and is a crucial vector of chikungunya virus in the subtropics and tropics [1–6]. Bhatt et al. [7] estimated that up to 390 million DENV infections, including close to 100 million cases of dengue disease manifestations, occur annually worldwide. *Aedes aegypti* originated in Africa, where its ancestral form was a zoophilic tree hole mosquito *Ae. aegypti formosus* [8]. The “domesticated” form of *Ae. aegypti* is genetically distinct with discrete geographical niches [9]. A study hypothesized that harsh conditions coupled with the slave trade resulted in the introduction of *Ae. aegypti* from Africa to the New World, from where it subsequently spread globally to tropical and subtropical regions [8]. Dengue fever is a travel-related disease in Taiwan because travelers can carry DENV from endemic areas into the island [10–14]. After being transported to the island, this virus is passed on to *Aedes* mosquitoes, which can result in local dengue fever outbreaks. During 1987–2002, the epidemiological patterns of dengue in Taiwan cycled with small-scale outbreaks occurring nearly every three years and large-scale epidemics occurring nearly every ten years [15]. However, according to the data of the Taiwan Centers for Disease Control (CDC), since 2002, nine outbreaks have been recorded with over 1000 cases of dengue fever, dengue hemorrhagic fever, and dengue shock syndrome [15]. *Aedes aegypti* in Southern Taiwan was closely associated with a high incidence of autochthonous dengue [16].

The geographical range of *Ae. aegypti* is considered to be approximately within low-latitude equatorward areas with an average winter isotherm of 10 °C in the northern and southern hemispheres [17, 18]. The core distributional areas of *Ae. aegypti*, where the mosquito and its associated pathogens present a major threat to human health, include regions of South Asia (particularly India, Sri Lanka and Bangladesh), regions of East Asia (particularly Southern China and Taiwan), Southeast Asia, Northeastern Australia, islands in the tropical Pacific Ocean, the subtropical and tropical parts of Africa, the Caribbean islands, and large parts of the Americas with cool range margins to the North and South [18]. Taiwan is geographically located in a region that spans both tropical and subtropical climates (22–25°N and 120–122°E). The latitude of 23.5°N divides the island into two climatic zones: (i) a tropical monsoon climate in the South and (ii) a subtropical monsoon climate in the North. The latitude, topography, ocean currents, and prevailing East Asian summer monsoon over Taiwan contribute to the island’s high temperature, humidity, and rainfall, as well as tropical cyclones during summer [19]. The magnitude of the effect of increasing temperatures on the seasonality and peak abundance of mosquitoes would likely vary over the climate suitability gradient across which *Ae. aegypti* occurs [20]. *Aedes aegypti* appears only south of 23.5°N and the Penghu Islet (i.e. Penghu County) [21]. In addition, the Taiwan CDC reported similar results based on surveys of the dengue vector *Ae. aegypti* conducted in Taiwan during 1982, 1988–1996, 2003–2005 and 2009–2011 [22]. The temporal distribution of *Ae. aegypti* was positively associated with rainfall and temperature; although summer is a rainy season and winter is a dry season, the occurrence of *Ae. aegypti* can be observed in each season [21, 23]. Moreover, predicting the effect of changing rainfall patterns on these mosquitoes is difficult because of the complicating factor that immature forms of *Ae. aegypti* are found in various containers, many of which are kept filled primarily by human action rather than rainfall [24]. Therefore, in Taiwan, we consider temperature to be a prominent and significant meteorological factor affecting ecological distributions of *Ae. aegypti*.

Here, we proposed that a critical low winter temperature prevents the expansion of *Ae. aegypti*. Data on the survival-limiting temperature and development rate of *Ae. aegypti* are commonly obtained from laboratory experiments under artificial feeding conditions. In Taiwan, studies have estimated the developmental zero water temperatures for larvae to be from 13 to 13.9 °C; studies have also indicated that *Ae. aegypti* immature forms were sensitive to lower winter temperatures in the north, resulting in their limited distribution in Taiwan [25, 26]. However, supporting evidence based on a large-scale field study is still insufficient, and particular threshold temperatures for the continued absence of *Ae. aegypti* in Northern Taiwan remain unclear. Therefore, the goal of the current study was to estimate the critical low temperature of *Ae. aegypti* by using data obtained from a three-year Taiwan CDC project conducted for national entomological surveillance and using nighttime land surface temperatures (LSTs) obtained from moderate resolution imaging spectroradiometer (MODIS) observations throughout the main island of Taiwan.

## Methods

### Research flowchart

We hypothesized that a particular threshold temperature in winter is highly correlated with the spatial distribution of *Ae. aegypti* and that this low critical temperature limits the distribution of *Ae. aegypti* in Taiwan.

**Fig. 1:**
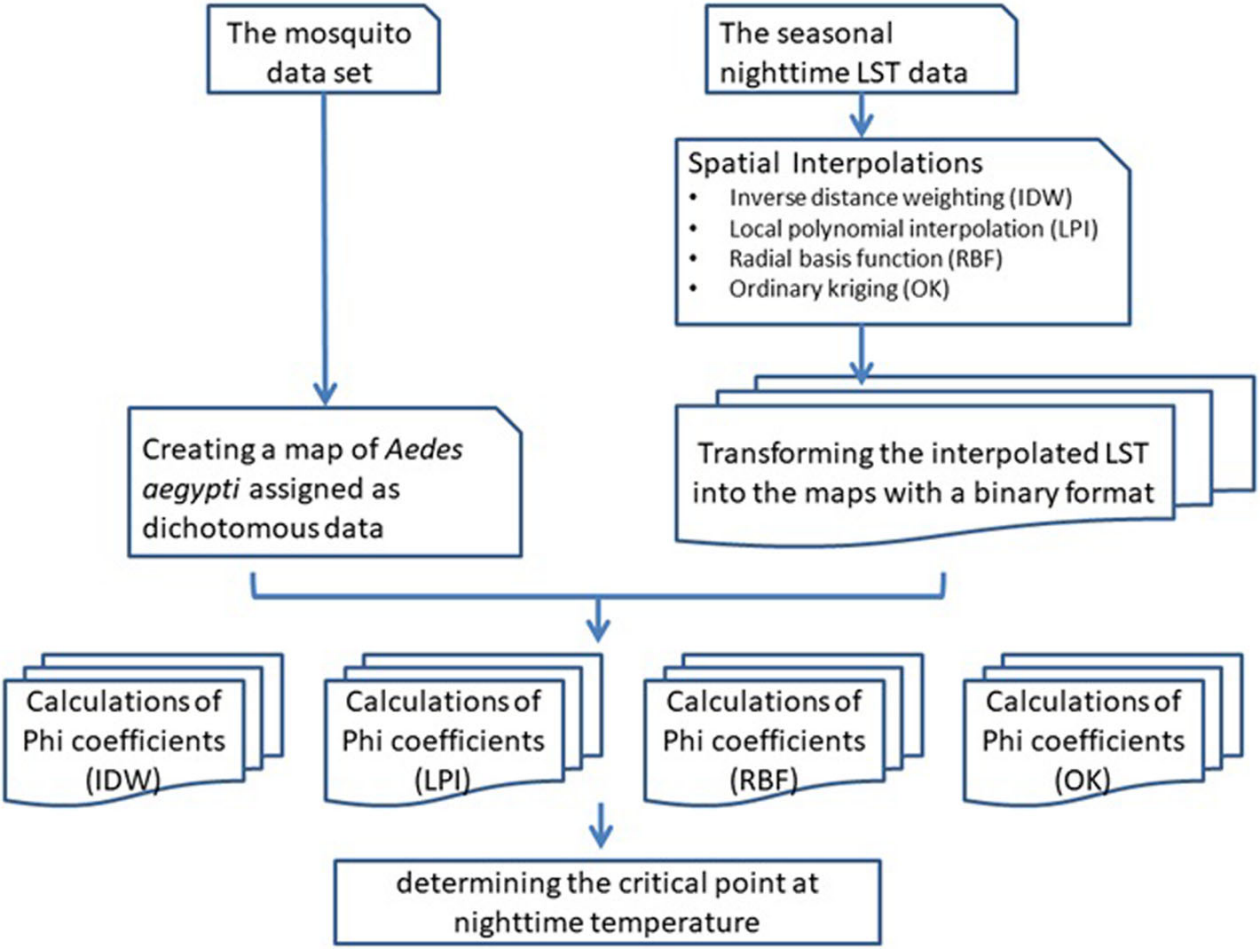
Research flowchart

The research flowchart is shown in Fig. 1. The mosquito dataset was transformed and assigned as a dichotomous dataset, which was based on the surveillance of *Ae. aegypti* in each of 348 townships in Taiwan. Even in MODIS monthly composites, 0–15% of the pixels could be missing because of clouds or other reasons. Therefore, the seasonal MODIS LST data in our study were processed to fill the missing data by using four interpolation methods [i.e. inverse distance weighting (IDW), local polynomial interpolation (LPI), radial basis function (RBF), and ordinary kriging (OK)]. According to the Taiwan Central Weather Bureau, minimum air temperatures during the period of 1981–2010 ranged between 12.4–18.1 °C in January and 25.1–26.4 °C in July in the plain areas of the main island of Taiwan [23]. Therefore, we assumed that the search for the low critical temperature should include a range from 12.4 to 26.4 °C. Consequently, we chose to include temperatures ranging from 8 to 28 °C. The interpolated LSTs are continuous data and must be converted into a dichotomous format for evaluating spatial similarity. The degree of the correlation between the geographical distribution of *Ae. aegypti* and LSTs can be evaluated by using phi coefficient calculations. The critical low temperature was defined by determining the highest phi coefficient at which the assumed temperature demonstrates a strong correlation with the spatial distribution of *Ae. aegypti*.

### Surveillance of *Aedes* mosquitoes on the main island of Taiwan

**Fig. 2:**
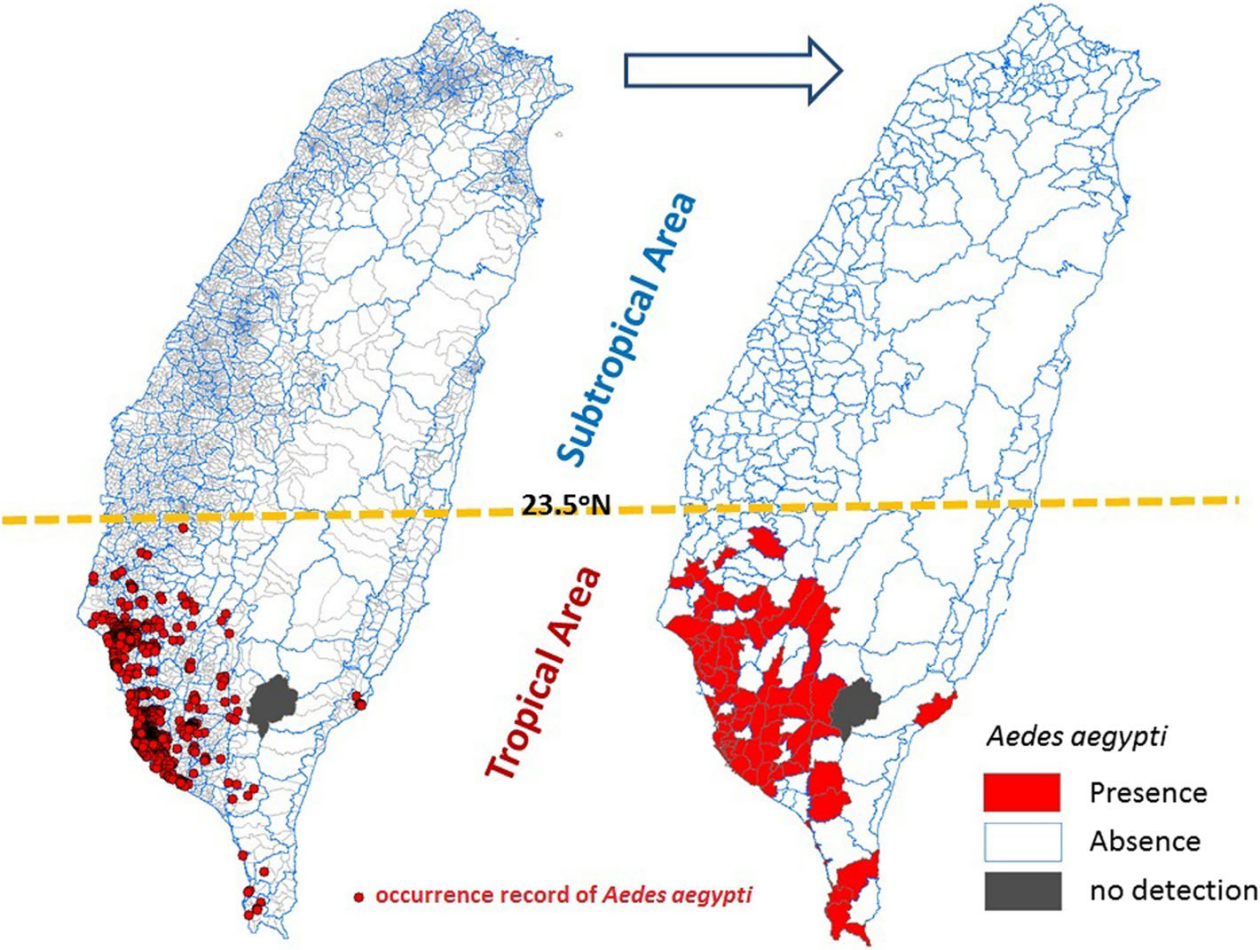
Map of 973 (among 7019) townships identified as having the presence of *Aedes aegypti*. These villages were then transformed so that they constituted 73 of the 348 townships investigated in this study

The mosquito dataset used in this study was a part of a national routine entomological surveillance of dengue, and the original data were collected in a 3-year project of the Taiwan CDC from 2009 to 2011 [22]. Briefly, 99.2% of a total of 368 townships and 91.1% of a total of 7835 villages completed this survey. The mean number of villages per township was 21 (range, 6–42 villages). Moreover, 3401, 2671 and 2423 villages were inspected in 2009, 2010 and 2011, respectively. The average area in each village was determined to be 4.6 km^2^ (range, 0.3–26.2 km^2^). A stratified cluster sampling technique was used to sample 50 residential premises in each village by randomly selecting and surveying the first house and then inspecting surrounding residences, checking water-filled containers indoors and outdoors. The containers included water buckets, pottery pots, urns, flower vases, flower saucers, used tires, tanks, gutters, discarded containers, natural containers, and other water-holding containers. *Aedes* larvae or pupae in containers were collected until the number reached 100. Then, samples were preserved in 70% alcohol and sent to the CDC for species identification. If the number after identification did not reach 100 due to misidentification of immature mosquitoes on site or because of low density, additional visits were conducted for that village. All mosquito data from the villages were categorized into 365 townships. In this study, we focused on the main island of Taiwan to detect spatial contiguity and pattern consistency; hence, townships belonging to outlying islands were excluded, and a total 348 townships were investigated (Wutai Township, which is located in a remote mountainous area, was the only township in the main island of Taiwan that was not investigated). We then mapped the distribution of *Ae. aegypti* by using the binary principle. A township was denoted as having “presence of *Ae. aegypti*” (defined as “1”) if it had one or more than one confirmed cases of *Ae. aegypti*; by contrast, a township was denoted as having the “absence of *Ae. aegypti*” (defined as “0”) if it had no confirmed cases of *Ae. aegypti*. Of the 7019 villages surveyed, 973 were identified as having *Ae. aegypti* presence. These villages were then transformed such that they constituted 73 of the 348 townships investigated in this study (Fig. 2).

### Satellite-derived LST products

**Fig. 3:**
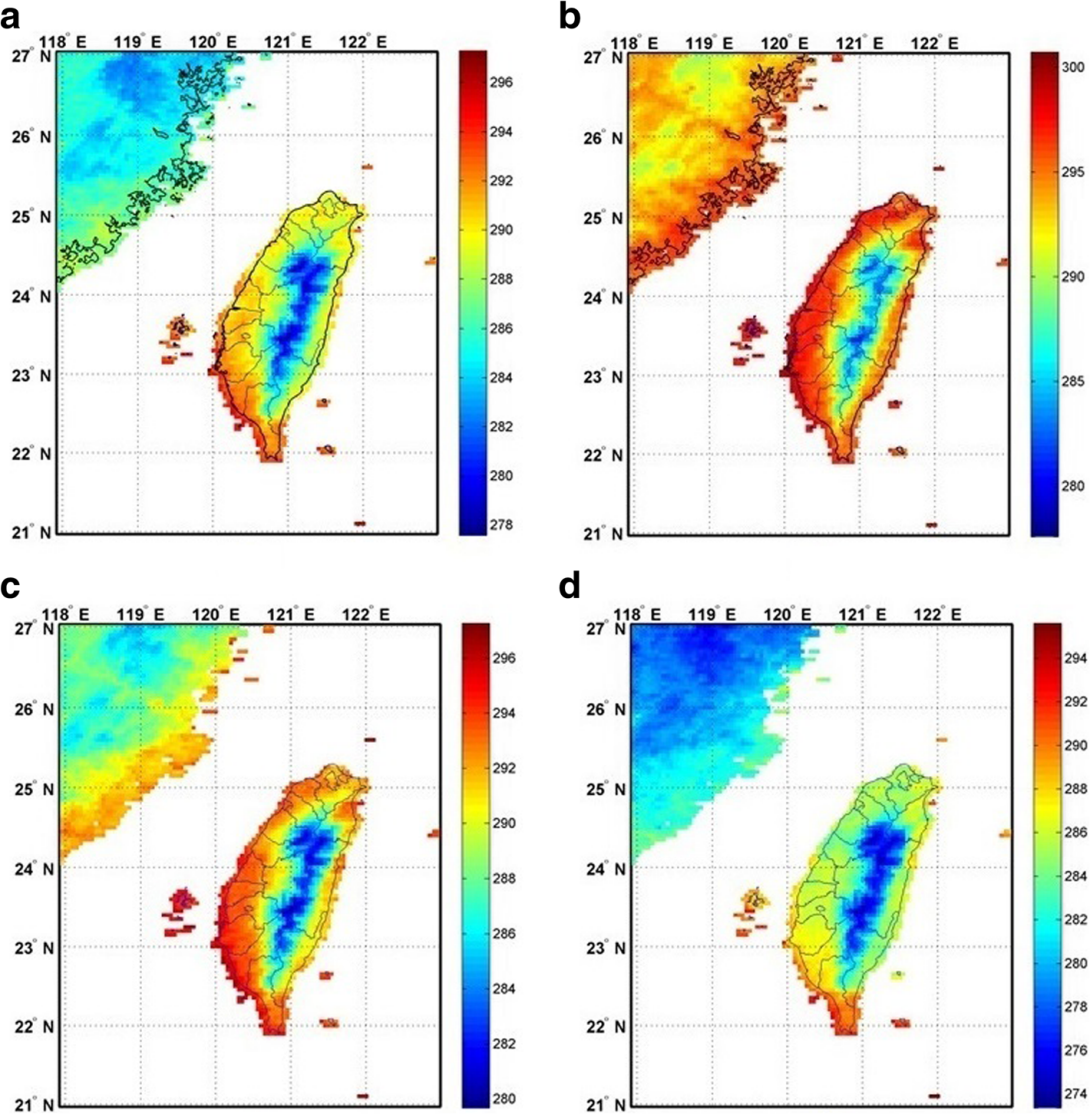
Average nighttime land surface temperature (LST) in spring (**a**), summer (**b**), autumn (**c**) and winter (**d**) from monthly LSTs obtained using the moderate resolution imaging spectroradiometer (MYD11C3) during the study period (unit in K)

The MODIS sensors onboard the Terra and Aqua satellites, two Earth Observing System platforms, can provide daily observations of terrestrial, atmospheric, and oceanographic variables on a global scale. Thus, MODIS products are widely used in meteorological studies worldwide, including LST data and images from MODIS thermal bands distributed by the Land Processes Distributed Active Archive Center of the US Geological Survey [27]. The MODIS LST and emissivity products can provide per-pixel temperature and emissivity values in a sequence of swath-to-grid-based global products. Both Aqua and Terra (MYD11 and MOD11) products contain Level 2 and 3 LST and emissivity retrieved from MODIS data at spatial resolutions of 0.01° [27] and 0.05° over global land surfaces under clear-sky conditions. The nominal accuracy of the MODIS LST product has been reported to be ±1 K. Some validation studies have reported accuracy statistics smaller than 1 K under clear-sky conditions within the temperature range of -10 to 50 °C [28]. In this study, nighttime LST retrievals on a 0.05° latitude/longitude climate modeling grid were collected and analyzed to determine covariates that were exhaustively known over the spatial-temporal domain in order to obtain complete prediction over a land mass. In MODIS monthly composites, 0–15% of the pixels are missing because of clouds or other factors. In this study, these missing pixels were replaced using four spatial interpolation models (i.e. IDW, LPI, RBF, and OK), which filled gaps in areas with sparse or irregularly spaced data points. The units in the original monthly MODIS LST images were also converted from Kelvin to degrees Celsius. The nighttime MODIS LST data were used to calibrate the entire study area by using spatial interpolation models (Fig. 3).

### Spatial interpolation

Spatial interpolation is the procedure of estimating the values of an environmental variable (e.g. nighttime LST) at locations with unknown values by using a sample of locations with known values. We employed spatial interpolation involving the application of four interpolation methods to a set of points with known values to create a continuous surface. These points are also known as sample points or observations.

### Inverse distance weighting

IDW estimates a value of each location by taking the distance-weighted average of the values of sample points in its neighborhood. The closer a sample point is to the location being estimated, the more influence or weight it has in the averaging process [29]. Mathematically, IDW can be expressed as follows:

$$ {Z}_0={\sum}_{i=1}^n{w}_i{Z}_i $$

$$ {w}_i=\frac{d_i^r}{\sum_{j=1}^n\left(1/{d}_i^r\right)} $$

where *Z*_0_ is the interpolated value of the point being estimated, *n* is the number of sample points in the neighborhood, *Z*_*i*_(*i* = 1, …, *n*) is the value of the *i*th sample point in the neighborhood, *d*_*i*_(*i* = 1, …, *n*) is the distance from the point being estimated to the *i*th sample point in the neighborhood, and *γ* is the power.

The power parameter determines the significance of sample points on the interpolated value. When a higher power is defined, more emphasis is placed on nearby points, producing a more varying and less smooth surface. Specifying a lower power places more emphasis on distant points, resulting in a smoother surface. A power of 2 is most commonly used with IDW [29].

### Local polynomial interpolation

A trend surface interpolation entails fitting a smooth surface defined by a polynomial function to a set of sample points and then using the polynomial function to estimate the values of unsampled locations. The general equation of a trend surface is as follows:

$$ f\left(x,y\right)={\sum}_{i,j=0}^p{b}_{i,j}{x}^i{y}^i $$

where *p* is the degree of the polynomial. As the polynomial order increases, the surface being fitted has an increasing number of curvatures and becomes progressively more complex. Although polynomial orders as high as 10 are accepted, numerical instability in the analysis often creates artifacts in the trend surfaces of orders higher than 5 [29].

We considered the simplest form of a trend surface to be a planar surface with no curvature, which is defined by a local linear or first-order polynomial:

$$ f\left(x,y\right)={b}_{0,0}+{b}_{1,0}x+{b}_{0,1}y $$

where *x* and *y* are coordinates, *b*_*i*, *j*_(*i*, *j* = 0, 1) are polynomial coefficients, and *f*(*x*, *y*) is the value of an environmental variable at the location (*x*, *y*). The coefficients of the polynomial function can be determined from sample points by minimizing

$$ {\sum}_{i=1}^n{w}_i{\left\{{z}_i-f\left({x}_i,{y}_i\right)\right\}}^2 $$

This is called a local least-squares method, which ensures that the sum of the squared deviations of observed values at sample points from the trend surface is a minimum. Suppose that there exist *n* sample points, whose values are *z*_1_, *z*_2_, …, *z*_*n*_ and the corresponding coordinates are (*x*_1_, *y*_1_), (*x*_2_, *y*_2_), …, (*x*_*n*_, *y*_*n*_); *w*_*i*_is a kernel smoothing weight and is given by a negative exponential function of the following form:

$$ {w}_i\left\{\begin{array}{c}{e}^{-{d}_i},{d}_i\le {d}_0\\ {}0,{d}_i>{d}_0\end{array}\right. $$

where *d*_*i*_(*i* = 1, …, *n*)is the distance from the point being estimated to the *i*th sample point; *d*_0_ is the bandwidth [30].

### Radial basis function

RBFs are conceptually similar to the approach of fitting a rubber membrane through measured sample values while minimizing the total curvature of the surface. An RBF is a real-valued function whose value depends only on the distance from the origin; the basic idea is to choose a radially symmetric function, φ(*r*_*i*_). The basis function selected determines how a rubber membrane would fit between the values [31]. The general form of an RBF can be written as

$$ {Z}_p={\sum}_{i=1}^n{w}_i\varphi \left({r}_i\right)+m $$

where *Z*_*p*_is the estimated value for the surface at the grid point *p*; φ(*r*_*i*_) is the RBF selected, with *r*_*i*_ being the radial distance from point *p* to the *i*th data point; and *w*_*i*_ is the weight and *m* the bias value (or Lagrangian multiplier), which are estimated from data points [32].

The form of the RBF in this study was a weighted regularized spline, which is expressed as follows:

$$ \upvarphi (r)=\mathit{\ln}{\left( cr/2\right)}^2+{E}_1{(cr)}^2+\gamma $$

where *γ* is the distance between the point and the sample, *c* is a smoothing parameter, and *γ* is Euler’s constant (*γ* = 0.577).

*E*_1_() is an exponential integral function given by

$$ {E}_1(x)=\underset{1}{\overset{\infty }{\int }}\frac{e^{- tx}}{t} dt $$

In the regularized type, the predicted surface becomes increasingly smooth as the weight value increases [31].

### Ordinary kriging

Kriging assumes that in most cases, spatial variations observed in environmental phenomena are random yet spatially correlated, and data values characterizing such phenomena conform to Tobler’s law of geography (data values at locations that are close to each other generally exhibit lower variability than data values at locations that are farther away from each other). This is called spatial autocorrelation. The exact nature of spatial autocorrelation varies with dataset and each dataset has its own unique function of variability and distance between sample points. This variability is represented by a semivariogram. Kriging uses semivariance values obtained from the fitted semivariogram to estimate the weights used in the interpolation and variance of interpolated values [29].

Semivariance measures the variability of observed values at sample points that are separated by a certain distance. It is calculated using the following equation:

$$ \gamma (h)=\frac{1}{2n}{\sum}_{i=1}^n{\left[z\left({x}_i+h\right)-z\left({x}_i\right)\right]}^2 $$

where *γ*(*h*) is the semivariance for a distance *h* separating two sample points *z*(*x*_*i*_) and *z*(*x*_*i*_ + *h*), and *n* is the number of pairs of sample points separated by *h*.

OK assumes that no trend exists in the data and that the mean of the dataset is unknown. The weights are derived by solving a system of linear equations, which minimizes the expected variance of data values:

$$ {\sum}_{j=1}^k{w}_j\gamma \left({h}_{ij}\right)+\mu =\gamma \left({h}_{i0}\right)\ \mathrm{for}\ \mathrm{all}\ i=1,\dots, n $$

$$ {\sum}_{i=1}^k{w}_i=1 $$

where *k* is the number of sample points within the neighborhood, *w*_*i*_ is the weight for the *i*th sample point to be estimated, *γ*(*h*_*ij*_) is the semivariance between sample points *i* and *j*, *γ*(*h*_*i*0_) is the semivariance between sample point *i* and the point to be estimated, and *μ* is a Lagrange multiplier, which is added to ensure the minimum possible estimation error. Once *w*_*i*_(*i* = 1, …, *k*) are found, the equation $\left({w}_i=\frac{d_i^r}{\sum_{j=1}^n\left(1/{d}_i^r\right)}\right)$ is used to estimate values at unsampled locations.

We selected the spherical semivariogram model to estimate the semivariogram that was fitted with a continuous curve.

The error variance for each interpolated point can be estimated using the following equation:

$$ {\sigma}^2={\sum}_{i=1}^k{w}_i\gamma \left({h}_{i0}\right)+\mu $$

The square root of the variance provides the standard error at the interpolated point, which yields an error estimate and the confidence interval for the unknown point.

Suppose the interpolated value is *z*_0_. If interpolation errors have a normal distribution, the real value at the interpolated point is within ${z}_0\pm \left(\sqrt{\sigma^2}\times 2\right)$ with a probability of 95% [29].

### Cross-validation

The cross-validation technique was adopted in this study to evaluate and compare the performance of different interpolation methods. The sample points were arbitrarily divided into two datasets, with one set used to train a model and the other used to validate the model. To reduce variability, the training and validation sets must cross over in successive rounds such that each data point could be validated against. The root mean square error (RMSE) for error measurement was estimated to evaluate the accuracy of the interpolation methods [33].

$$ \mathrm{RMSE}=\sqrt{\frac{\sum_{i=1}^N{\left({O}_i-{P}_i\right)}^2}{N}} $$

where *O*_*i*_ is the observed value, *P*_*i*_ was the predicted value, and *N* is the number of samples.

Seasonal data derived from MODIS raster images were evaluated using the four spatial interpolation methods (i.e. IDW, LPI, RBF, and OK). The interpolation methods were used to transform point data into zonal data by using ArcMap 10, and each model automatically generated the minimum, mean, and maximum estimates. The spatial interpolation models and error measurements (i.e. RMSE) applied in this study were mapped using ArcMap 10.

### Transformation of the interpolated LST into 348-township maps by using the binary principle

We assumed that a critical low temperature limits the biogeographical distribution of *Ae. aegypti* to Southern Taiwan. Designing criteria for creating a binary map of 384 townships for LSTs was necessary; from a binary map, the extent of spatial similarity could be evaluated by comparing geographical distributions between *Ae. aegypti* and LSTs. The threshold temperatures were estimated to range from 8 to 28 °C, encompassing the seasonal temperatures in the plain areas of Taiwan. Criteria were designed as follows: (i) if the LST of a township was higher than an assumed temperature, then this location was considered a favorable habitat for the survival of *Ae. aegypti* (optimal survival conditions); (ii) if the LST of a township was lower than a given temperature assumption, then this location was considered harmful for the survival of *Ae. aegypti* (nonoptimal survival conditions); and (iii) according to the binary principle, optimal and nonoptimal survival conditions were defined as “1” and “0,” respectively. Scenarios from each of the given temperature assumptions were generated, which were then mapped to a dichotomous map of the 348 townships.

### Phi coefficient

The phi coefficient (∅) is the version of Pearson’s *γ* that is used when both X and Y are true dichotomous variables [34]. It can be calculated from general Pearson’s *γ* by using score values of 0 and 1 or 1 and 2 for group membership variables. Alternatively, the phi coefficient can be computed from cell frequencies in a 2×2 table that summarizes the number of cases for each combination of X and Y scores. The frequencies of cases in the four cells of a 2×2 table are labeled in order to compute the phi coefficient from the cell frequencies. Assuming that the cell frequencies are from a to d (i.e. *a* and *d* correspond to “discordant” outcomes and *b* and *c* correspond to “concordant” outcomes), then the following formula can be used to compute the phi coefficient directly from the cell frequencies:

$$ \varnothing =\frac{bc- ad}{\sqrt{\left(a+b\right)\times \left(a+c\right)\times \left(b+d\right)\times \left(c+d\right)}} $$

where *b* and *c* are the numbers of cases in the concordant cells of a 2×2 table and *a* and *d* are the numbers of cases in the discordant cells of a 2 × 2 Table.

A formal significance test for the phi coefficient can be obtained by converting it into a chi-square; in the following equation, *N* represents the total number of scores in the contingency table:

$$ \varnothing =\sqrt{\frac{{\mathrm{X}}^2}{N}} $$

This is a chi-square statistic with a degree of freedom (*df*) of 1. This can be used as one of the many possible statistics to describe relationships between categorical variables based on the tables of cell frequencies. For *X*^2^ with *df* = 1 and α = 0.05, the critical value of *X*^2^ is 3.84; thus, if the obtained *X*^2^ value exceeds 3.84, then the phi coefficient is statistically significant at the 0.05 level [34].

In this study, a correlation statistic was computed using the phi coefficient to evaluate the correlation of the binary variables, namely *Ae. aegypti* geographical distribution and LST, under a given temperature assumption (details described in “Transformation of the interpolated LST into 348-township maps by using the binary principle” in the Methods section). Phi coefficient statistics were calculated using SPSS 12 (SPSS, Chicago, IL, US).

## Results

The results of the RMSE test are presented in Table 1. The RMSE values obtained when fitting the IDW model to the seasonal LSTs were 0.51, 0.6, 0.53 and 0.55 in spring, summer, autumn and winter, respectively. The RMSE values obtained when fitting the LPI model to the seasonal LSTs were 0.44, 0.53, 0.46 and 0.47 in spring, summer, autumn and winter, respectively. The RMSE values for fitting the RBF model were 0.47, 0.51, 0.48 and 0.48 in spring, summer, autumn and winter, respectively. The RMSE values for fitting the OK model were 0.4, 0.48, 0.42 and 0.44 in spring, summer, autumn and winter, respectively. All RMSE values were lower than 0.6, indicating that the deviation of the interpolated estimation was in the range of ± 0.6 °C. The OK model, which had the lowest RMSE values among the four interpolation models in all four seasons, was determined to be an optimal model for processing the nighttime LST data.

**Table 1:** Descriptive statistics of the root mean square error in cross-validation of the studied interpolation models. The sample number of observations from moderate resolution imaging spectroradiometer (MODIS) was 3215. Four seasons were defined: spring (from March to May), summer (from June to August), autumn (from September to November), and winter (from December to February)

Season	IDW	LPI: exponential kernel function	RBF: completely regularized spline	OK: spherical type
Spring	0.51	0.44	0.47	0.40
Summer	0.60	0.53	0.51	0.48
Autumn	0.53	0.46	0.48	0.42
Winter	0.55	0.47	0.48	0.44

**Fig. 4:**
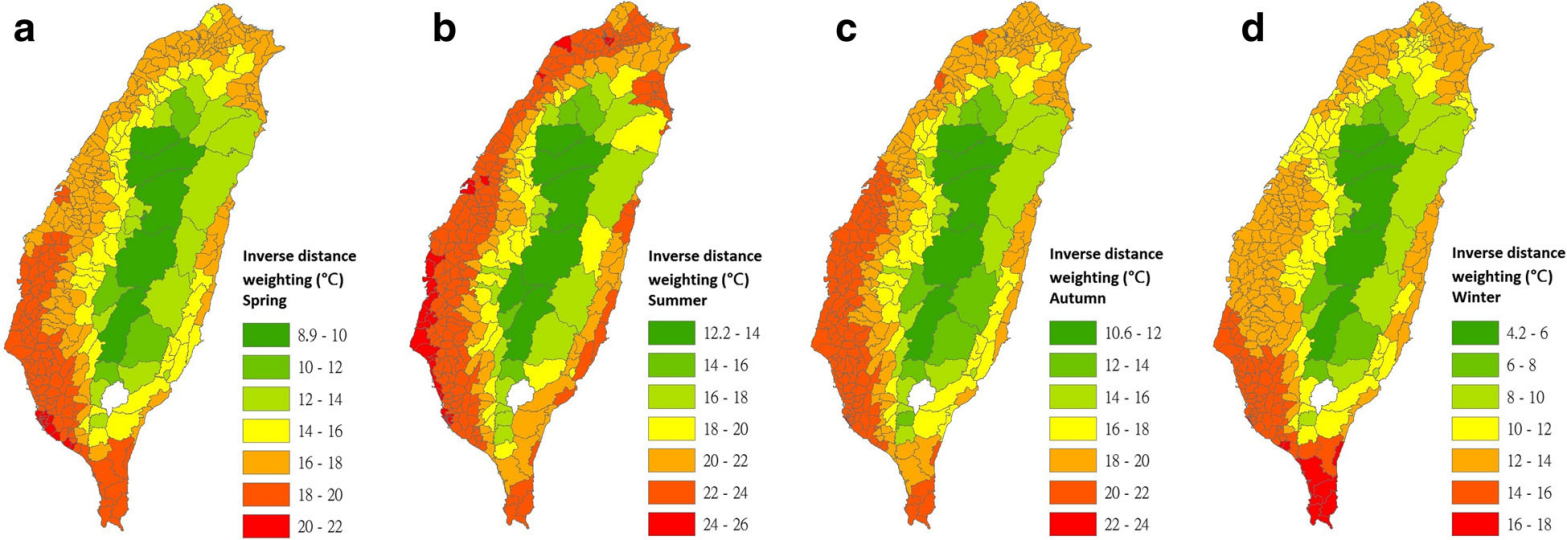
Maps of nighttime land surface temperatures in 348 townships for each season that were interpolated using the inverse distance weighting model (2009–2011). **a** Interpolated estimates for spring. **b** Interpolated estimates for summer. **c** Interpolated estimates for autumn. **d** Interpolated estimates for winter

**Fig. 5:**
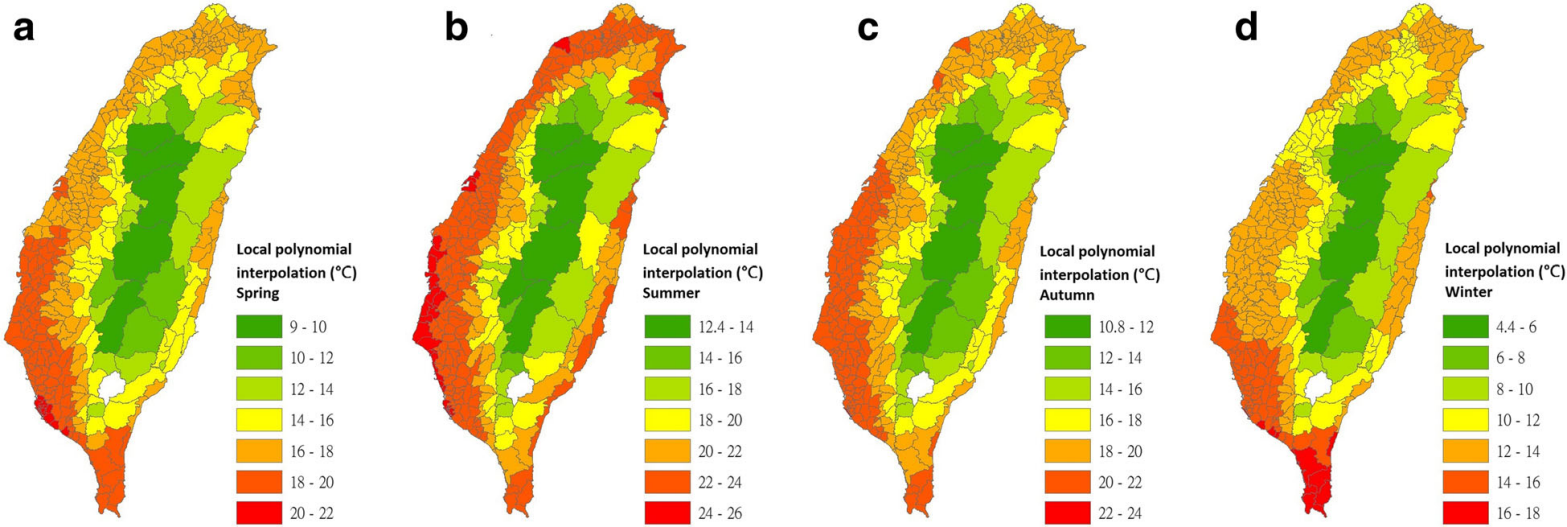
Maps of nighttime land surface temperatures in 348 townships for each season that were interpolated using the local polynomial interpolation model (2009–2011). **a** Interpolated estimates for spring. **b** Interpolated estimates for summer. **c** Interpolated estimates for autumn. **d** Interpolated estimates for winter

**Fig. 6:**
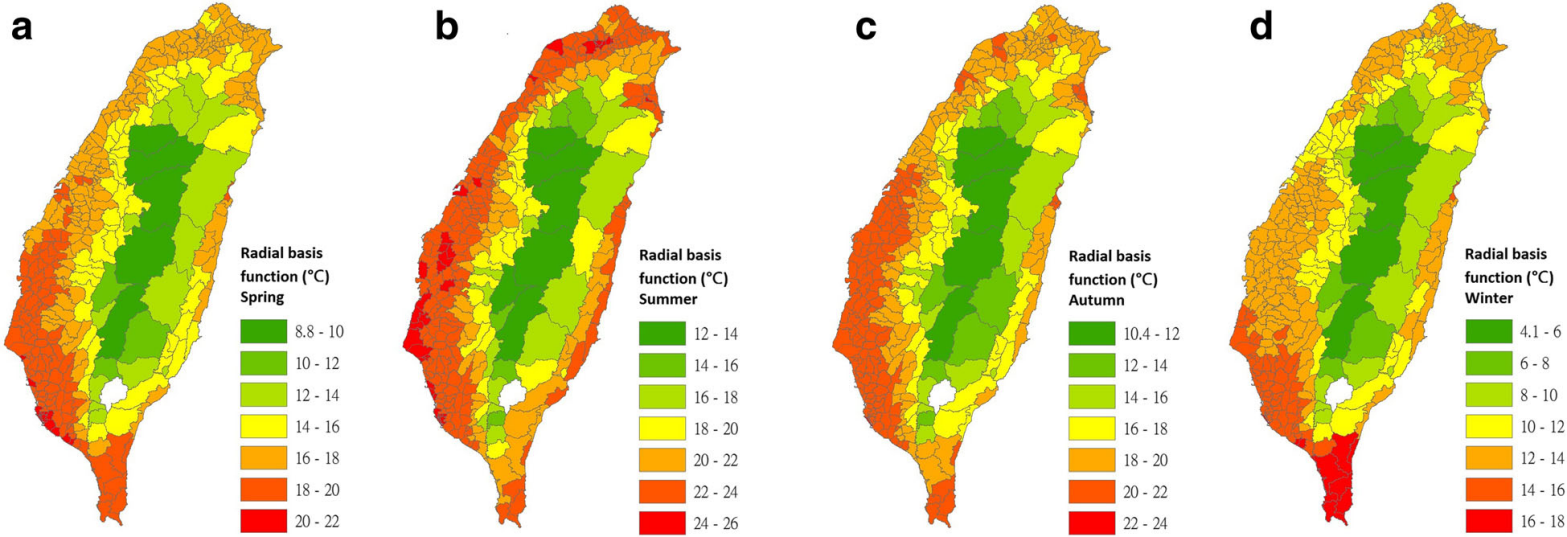
Maps of nighttime land surface temperatures in 348 townships for each season that were interpolated using the radial basis function model (2009–2011). **a** Interpolated estimates for spring. **b** Interpolated estimates for summer. **c** Interpolated estimates for autumn. **d** Interpolated estimates for winter

**Fig. 7:**
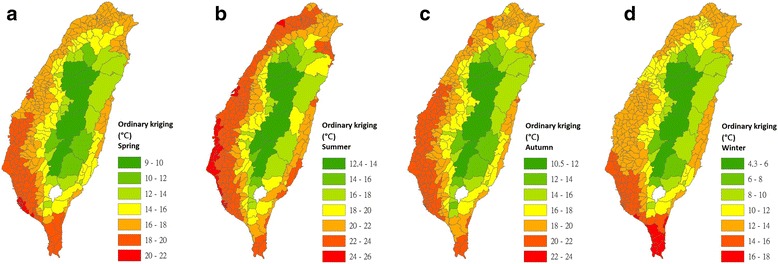
Maps of nighttime land surface temperatures in 348 townships for each season that were interpolated using the ordinary kriging model (2009–2011). **a** Interpolated estimates for spring. **b** Interpolated estimates for summer. **c** Interpolated estimates for autumn. **d** Interpolated estimates for winter

Maps of nighttime LSTs in the 348 townships of Taiwan for the four seasons were interpolated using the IDW (Fig. 4), LPI (Fig. 5), RBF (Fig. 6) and OK (Fig. 7) models.

**Fig. 8:**
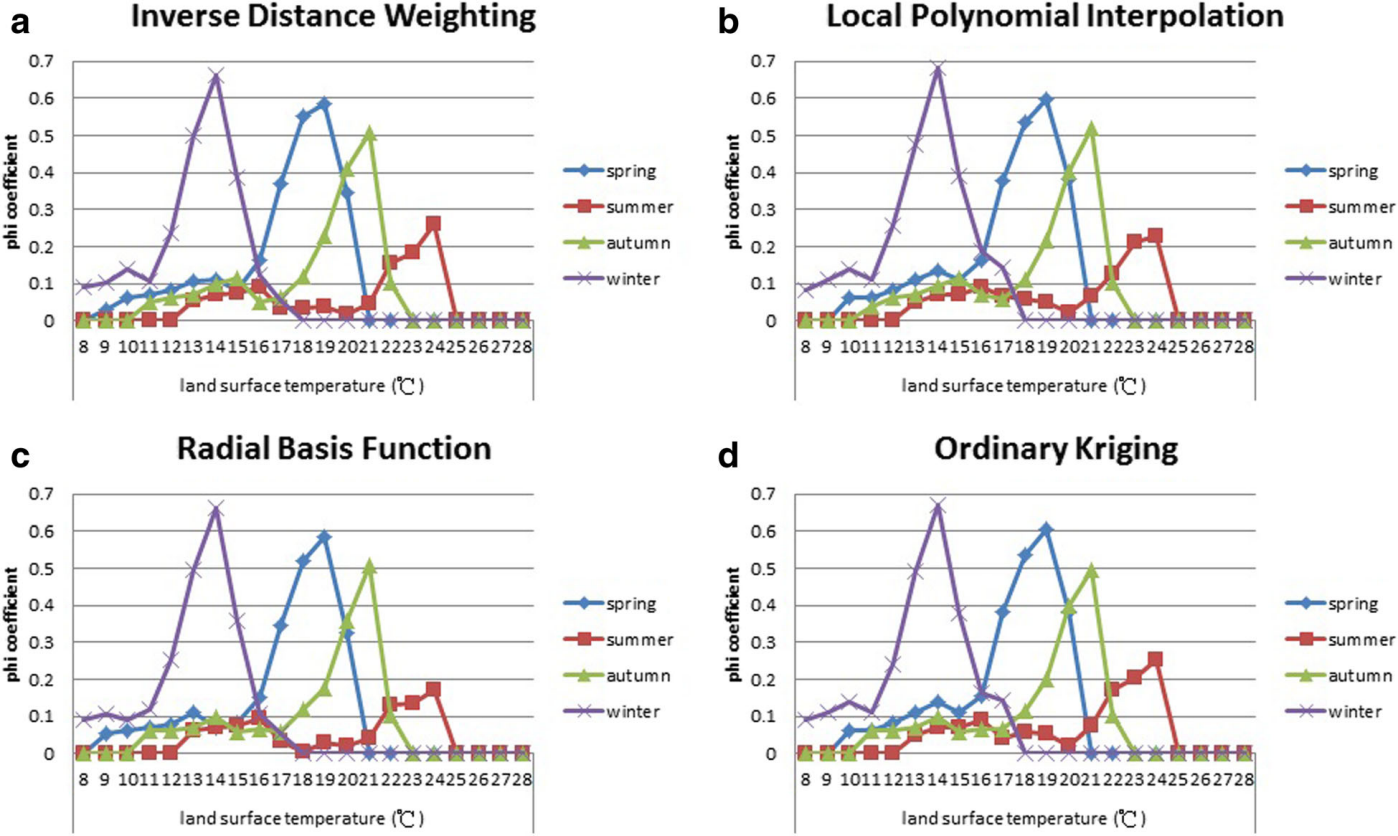
Phi coefficients indicating the correlation between the geographical distributions of *Aedes aegypti* and temperature assumptions based on a 348-township binary map for each season. **a** Spatial interpolation models calculated using inverse distance weighting. **b** Spatial interpolation models calculated using local polynomial interpolation. **c** Spatial interpolation models calculated using radial basis function. **d** Spatial interpolation models calculated using ordinary kriging

A phi coefficient statistic was applied to calculate the correlation between the geographical distribution of *Ae. aegypti* and nighttime LST assumptions. The threshold temperature assumptions were made by following the criteria mentioned in the Methods section (Transformation of the interpolated LST into 348-township maps by using the binary principle). The threshold temperatures were estimated to range from 8 to 28 °C with an interval of 1 °C. We considered that a particular threshold temperature limits the distribution of *Ae. aegypti* in Southern Taiwan, and such a threshold temperature was determined to be associated with the highest phi coefficient. The results obtained for the IDW model for all four seasons, indicated that the phi coefficient showed the highest peak value (∅ = 0.66 at 14 °C) in winter, the second highest peak value (∅ = 0.58 at 19 °C) in spring, the third highest peak value (∅ = 0.51 at 21 °C) in autumn, and the lowest peak value (∅ = 0.26 at 24 °C) in summer (Fig. 8a). A phi coefficient higher than 0.141 was considered to signify a significant correlation at the 0.05 level. For the LPI model, the phi coefficient showed the highest peak value (∅ = 0.68 at 14 °C) in winter, the second highest peak value (∅ = 0.60 at 19 °C) in spring, the third highest peak value (∅ = 0.52 at 21 °C) in autumn, and the lowest peak value (∅ = 0.23 at 24 °C) in summer (Fig. 8b). In addition, for the RBF model, the phi coefficient showed the highest peak value (∅ = 0.66 at 14 °C) in winter, the second highest peak value (∅ = 0.58 at 19 °C) in spring, the third highest peak value (∅ = 0.51 at 21 °C) in autumn, and the lowest peak value (∅ = 0.17 at 24 °C) in summer (Fig. 8c). Finally, for the OK model, the phi coefficient showed the highest peak value (∅ = 0.67 at 14 °C) in winter, the second highest peak value (∅ = 0.60 at 19 °C) in spring, the third highest peak value (∅ = 0.50 at 21 °C) in autumn, and the lowest peak value (∅ = 0.25 at 24 °C) in summer (Fig. 8d). The highest phi coefficients were consistent in all interpolation models and occurred in winter, indicating that the geographical distribution of *Ae. aegypti* is more strongly correlated with nighttime LSTs in winter than in other seasons.

In Taiwan, low temperature during winter is a critical factor affecting the geographical distribution of *Ae. aegypti*. The threshold temperature was determined and is shown in Table 2. According to the highest phi coefficient, which was used to determine the correlation between winter temperatures and the geographical distribution of *Ae. aegypti*, in the IDW model, the minimum, mean, and maximum temperatures that had the strongest correlation with the distribution of *Ae. aegypti* were 13.5 °C (∅ = 0.68), 13.8 °C (∅ = 0.71), and 14 °C (∅ = 0.69), respectively. In the LPI model, the minimum, mean, and maximum temperatures that had the strongest correlation with the distribution of *Ae. aegypt* were 13.8 °C (∅ = 0.7), 13.8 °C (∅ = 0.68), and 13.9 °C (∅ = 0.67), respectively. In the RBF model, the minimum, mean, and maximum temperatures that had the strongest correlation with the distribution of *Ae. aegypt* were 13.4 °C (∅ = 0.67), 13.8 °C (∅ = 0.69), and 14.1 °C (∅ = 0.7), respectively. Finally, in the OK model, the minimum, mean, and maximum temperatures that had the strongest correlation with the distribution of *Ae. aegypt* were 13.7 °C (∅ = 0.71), 13.8 °C (∅ = 0.69), and 14 °C (∅ = 0.71), respectively. Thus, based on different statistical models, the results revealed that the critical low temperature of *Ae. aegypti* in Taiwan was 13.8 °C (mean value) and ranged from 13.7 to 14 °C, according to the results of the OK interpolation method. A mean LST of 13.8 °C is a critical temperature to limit the occurrence of *Ae. aegypti* North of 23.5°N, the subtropical region of Taiwan, particularly during winter.

**Table 2:** Highest values of phi coefficients demonstrating correlation between *Aedes aegypti* geographic distribution and threshold temperatures. The temperature assumption is estimated following the criteria mentioned in Methods. The interval of temperature assumption is 0.1 °C

Interpolation techniques		Land surface night temperature (°C)		
		Minimum	Mean	Maximum
Inverse distance weighting	Threshold temperature	13.5	13.8	14.0
	The highest value of phi coefficient	0.68	0.71	0.69
Local polynomial interpolation: exponential kernel function	Threshold temperature	13.8	13.8	13.9
	The highest value of phi coefficient	0.70	0.68	0.67
Radial basis function: completely regularized spline	Threshold temperature	13.4	13.8	14.1
	The highest value of phi coefficient	0.67	0.69	0.70
Ordinary kriging: spherical type	Threshold temperature	13.7	13.8	14.0
	The highest value of phi coefficient	0.71	0.69	0.71

## Discussion

Developmental zero growth is based on a theoretical line, which establishes a gradient that corresponds to the growth rates at particular water temperatures in relation to immature insect forms. For *Ae. aegypti*, the relationship between water temperatures and development rates was similar for eggs, larvae, and pupae. Once the lower developmental zero temperature (10–14 °C) was exceeded, a near-linear relationship was observed up to 30 °C. Then, the development rate remained relatively stable or even decreased slightly before falling considerably near the upper developmental zero temperature, which occurred at 38–42 °C [20]. On the basis of the observed linear relationship between specific life stages and water temperatures, the lower developmental zero temperature has been estimated to be at 10–14 °C for eggs, 11.8–14 °C for larvae, and 10–12 °C for pupae [17, 20, 25, 35–39]. Based on the spatial similarity approach, our results indicate that the threshold land temperature ranged from 13.7 °C to 14 °C; this statistical finding is similar to those reported in previous studies evaluating the threshold water temperature for the eggs or larvae of *Ae. aegypti*. Moreover, the temperature extremes of 16 and 36 °C considerably decreased adult longevity and female fecundity [40]. Regarding survival time for females at extreme air temperatures, studies have reported that females uniformly survived 60 min of exposure to 39–40 °C; however, only 15–30 min of exposure to 42–43 °C resulted in 65% mortality, and 30 min of exposure to 45–51 °C was uniformly lethal [17, 41]. At extreme lower temperatures, adults could survive for 24–72 h when exposed to 4.4 °C [42] and for weeks at constant temperatures of 7–9 °C [43]. The effects of a diurnal temperature range (DTR) on female adult survival have also been shown to be considerable; an increasing DTR at a mean temperature of approximately 26 °C reduced the survival of females over an experimental period from 70% for a DTR of 0 °C to 50% for a DTR of 10 °C and 30% for a DTR of 20 °C [44]. Follow-up studies have also found that a DTR of 18.6 °C reduced female survival and reproductive output [45, 46].

In addition to temperature, water nutrient conditions, larval abundance, and the presence of biological competitors, such as *Ae. albopictus*, or predators also affect the growth and survival of *Ae. aegypti* larvae. Larvae reared in lower diet concentrations survived longer than those reared in higher diet concentrations [47]. Competition and succession in territories of coexistence between *Ae. aegypti* and *Ae. albopictus* have been reported [48–50]. However, *Ae. aegypti* tends to breed in urbanized ecological niches, whereas *Ae. albopictus* is more frequently found in rural areas [51]. *Ae. aegypti* prefers living indoors, whereas *Ae. albopictus* frequently breeds outdoors [52–55]. However, the apparent coexistence of the two species could be a transient situation, followed by a reduction [49, 56–60] or displacement [61, 62] of the resident species or satyrization [63, 64]. Furthermore, oocysts of *Ascogregarina* parasites (such as *As. taiwanensis* and *As. culicis*) can infect different stages of *Aedes* mosquito larvae, and oocyst dissemination can occur at the time of adult mosquito emergence and oviposition [65]. For example, the *Ascogregarina* parasite was detected in 16.7% of *Ae. aegypti* immature forms in temperate Argentina [66]. In the Amazon region of Brazil, the percentage of *Ae. albopictus* larvae infected by *As. taiwanensis* ranged between 21 and 93.5% and that of *Ae. aegypti* larvae infected by *As. culicis* ranged between 22 and 95% [67]. In Taiwan, field studies of *Ascogregarina* parasites in three coastal townships in Chiayi County (the area having the northernmost distribution of *Ae. aegypti* in Taiwan) reported that larvae of *Aedes* spp. could also be infected by *Ascogregarina* parasites, and the average infection rate was 4.2% in *Ae. aegypti* and 10.5% in *Ae. albopictus* in one 1994–1995 survey [68]. Socioeconomic factors affecting the distribution of *Aedes* mosquitoes, other than the use of containers to store water, include air conditioner use, housing quality, and urbanization rate [69, 70]. Extensive and rapid urbanization without appropriate planning may have directly resulted in large numbers of artificial containers (e.g. concrete tanks with broken lids) that are suitable for breeding *Aedes* mosquitoes in and around households [71–73].

According to reports from the Taiwan Malaria Research Institute, a few sporadic records of *Ae. aegypti* were documented in the townships of Northern Taiwan (i.e. Jhubei Township in Hsinchu County and Suao Township in Yilan County) and Central Taiwan (i.e. Nantou Township in Nantou County and a few townships in Yunlin County) during 1901–1964; however, *Ae. aegypti* never successfully established colonies in these townships [21]. As reported by the Central Weather Bureau [74], the surface air temperature in winter is cold in Northern Taiwan with a seasonal average of approximately 16 °C under the influence of the prevailing East Asian winter monsoon. Conversely, Southern Taiwan is very dry in winter with a seasonal mean rainfall of less than 50 mm, a trend that reverses in summer. During summer in Taiwan, the seasonal mean of the surface air temperature is nearly 28 °C, with the maximum temperature being more than 35 °C, which is magnified in urban areas, which are impacted by the East Asian Summer Monsoon, local circulation, and the urban heat-island effect. During the past century, the mean surface air temperature in Taiwan has increased by approximately 0.8 °C [74], and this may affect the distribution of *Ae. aegypti* in the future.

## Conclusions

The critical low temperature for the survival of *Ae. aegypti* was statistically analyzed in this study. The phi coefficient was used to evaluate the degree of association between the ecological distributions of *Ae. aegypti* and the spatial distribution of seasonal nighttime temperature assumptions. The highest phi coefficient was considered to be associated with the critical low temperature for immature forms of *Ae. aegypti*. An LST as low as 13.8 °C in winter was considered to be the critical temperature for *Ae. aegypti* larvae, which could result in near disappearance of *Ae. aegypti* in winter in the subtropical regions of Taiwan under the influence of the prevailing East Asian winter monsoon. Because of the major role of *Ae. aegypti* as a vector of human pathogens, it is imperative to investigate the extent to which climate change, particularly rising temperatures and changes in rainfall patterns, affects the geographical distribution, local seasonal activity pattern, and peak abundance of *Ae. aegypti*, as well as the risk of human DENV infection. Our data on LST obtained using satellite telemetry can be used in mosquito control and public health management planning in the future.

## References

[1] Gubler DJ (2004). The changing epidemiology of yellow fever and dengue, 1900 to 2003: full circle?. Comp Immunol Microbiol Infect Dis.

[2] Barrett AD, Higgs S (2007). Yellow fever: a disease that has yet to be conquered. Annu Rev Entomol.

[3] Pialoux G, Gaüzère BA, Jauréguiberry S, Strobel M (2007). Chikungunya, an epidemic arbovirosis. Lancet Infect Dis.

[4] Jentes ES, Poumerol G, Gershman MD, Hill DR, Lemarchand J, Lewis RF (2011). The revised global yellow fever risk map and recommendations for vaccination, 2010: consensus of the informal WHO working group on geographic risk for yellow fever. Lancet Infect Dis.

[5] Simmons CP, Farrar JJ, Chau NVV, Wills B (2012). Dengue. N Engl J Med.

[6] Leparc-Goffart I, Nougairede A, Cassadou S, Prat C, de Lamballerie X (2014). Chikungunya in the Americas. Lancet.

[7] Bhatt S, Gething PW, Brady OJ, Messina JP, Farlow AW, Moyes CL (2013). The global distribution and burden of dengue. Nature.

[8] Brown JE, Evans BR, Zheng W, Obas V, Barrera-Martinez L, Egizi A (2014). Human impacts have shaped historical and recent evolution in *Aedes aegypti*, the dengue and yellow fever mosquito. Evolution.

[9] Brown JE, McBride CS, Johnson P, Ritchie S, Paupy C, Bossin H (2011). Worldwide patterns of genetic differentiation imply multiple ‘domestications’ of *Aedes aegypti*, a major vector of human diseases. Proc Biol Sci.

[10] King CC, Wu YC, Chao DY, Lin TH, Chow L, Wang HT (2000). Major epidemics of dengue in Taiwan in 1981–2000: related to intensive virus activities in Asia. Dengue Bull.

[11] Shu PY, CL S, Liao TL, Yang CF, Chang SF, Lin CC (2009). Molecular characterization of dengue viruses imported into Taiwan during 2003–2007: geographic distribution and genotype shift. Am J Trop Med Hyg.

[12] Lin CC, Huang YH, Shu PY, HS W, Lin YS, Yeh TM (2010). Characteristic of dengue disease in Taiwan: 2002–2007. Am J Trop Med Hyg.

[13] Huang JH, CL S, Yang CF, Liao TL, Hsu TC, Chang SF (2012). Molecular characterization and phylogenetic analysis of dengue viruses imported into Taiwan during 2008–2010. Am J Trop Med Hyg.

[14] van Panhuis WG, Choisy M, Xiong X, Chok NS, Akarasewi P, Iamsirithaworn S (2015). Region-wide synchrony and traveling waves of dengue across eight countries in Southeast Asia. Proc Natl Acad Sci U S A.

[15] Taiwan National Infectious Disease Statistics System. Centers for Disease Control, R.O.C. (Taiwan). http://nidss.cdc.gov.tw/default.aspx. Accessed 16 May 2017.

[16] Tsai PJ, Teng HJ (2016). Role of *Aedes aegypti* (Linnaeus) and *Aedes albopictus* (Skuse) in local dengue epidemics in Taiwan. BMC Infect Dis.

[17] Christophers RS (1960). *Aedes aegypti* (L.), the yellow fever mosquito: its life history, bionomics and structure.

[18] World Health Organization. Dengue:guidelines for diagnosis, treatment. Prev Control. 2009; http://www.who.int/tdr/publications/documents/dengue-diagnosis.pdf. Accessed 16 May 2017 23762963

[19] McKnight TL, Hess D. Physical geography: a landscape appreciation. Upper Saddle River, NJ: Prentice Hall; 2000.

[20] Eisen L, Monaghan AJ, Lozano-Fuentes S, Steinhoff DF, Hayden MH, Bieringer PE (2014). The impact of temperature on the bionomics of *Aedes* (*Stegomyia*) *aegypti*, with special reference to the cool geographic range margins. J Med Entomol.

[21] Huang CC, Chen CS (1986). The distribution of *Aedes aegypti* and *Ae. albopictus* in Taiwan. Tunghai Biol.

[22] Lin C, Wang CY, Teng HJ (2014). The study of dengue vector distribution in Taiwan from 2009 to 2011. Taiwan. Epidemiol Bull.

[23] Central Weather Bureau. Climate Statistic. http://www.cwb.gov.tw/V7e/climate/monthlyMean/Precipitation.htm. Accessed 16 May 2017.

[24] Tun-Lin W, Lenhart A, Nam VS, Rebollar-Téllez E, Morrison AC, Barbazan P (2009). Reducing costs and operational constraints of dengue vector control by targeting productive breeding places: a multi-country non-inferiority cluster randomized trial. Tropical Med Int Health.

[25] Chen CS, Huang CC (1988). Ecological studies on *Aedes aegypti* and *Ae. albopictus* I. Comparison of development threshold and life tables. Yushania.

[26] Chang LH, Hsu EL, Teng HJ, Ho CM (2007). Differential survival of *Aedes aegypti* and *Aedes albopictus* (Diptera: Culicidae) larvae exposed to low temperatures in Taiwan. J Med Entomol.

[27] Coll C, Wan Z, Galve JM (2009). Temperature-based and radiance-based validations of the V5 MODIS land surface temperature product. J Geophys Res.

[28] Yoo JM, Won YI, Cho YJ, Jeong MJ, Shin DB, Lee SJ (2011). Temperature trends in the skin/surface, mid-troposphere and low stratosphere near Korea from satellite and ground measurements. Asia-Pac J Atmos Sci.

[29] Zhu X, Zhu X (2016). Spatial analysis. GIS for environmental applications: a practical approach.

[30] Smith TE (2016). Notebook on spatial data analysis.

[31] Liu JG, Mason PJ, Liu JG, Mason PJ (2009). Extracting information from point data: geostatistics. Essential image processing and GIS for remote sensing.

[32] de Smith M, Goodchild MF, Longley P. Radial basis and spline functions. In: Geospatial analysis. 5th ed. http://www.spatialanalysisonline.com/HTML/index.html?radial_basis_and_spline_functi.htm. Accessed 22 Apr 2017.

[33] Wise S (2014). GIS Fundamentals.

[34] Warner RM, Warner RM (2013). Alternative correlation coefficients. Applied statistics: from bivariate through multivariate techniques.

[35] Fielding JW (1919). Notes on the bionomics of *Stegomyia fasciata*, Fabr. Part I. Ann Trop Med Parasitol.

[36] Davis NC (1932). The effects of heat and of cold upon *Aedes* (*Stegomyia*) *aegypti*. Part I. The survival of *Stegomyia* eggs under abnormal temperature conditions. Am J Hyg.

[37] Bar-Zeev M (1958). The effect of temperature on the growth rate and survival of the immature stages of *Aedes aegypti* (L.). Bull Entomol Res.

[38] Tun-Lin W, Burkot TR, Kay BH (2000). Effects of temperature and larval diet on development rates and survival of the dengue vector *Aedes aegypti* in north Queensland, Australia. Med Vet Entomol.

[39] Couret J, Benedict MQ (2014). A meta-analysis of the factors influencing development rate variation in *Aedes aegypti* (Diptera: Culicidae). BMC Ecol.

[40] Costa EAPA, Santos EMM, Correia JC, Albuquerque CMR (2010). Impact of small variations in temperature and humidity on the reproductive activity and survival of *Aedes aegypti* (Diptera, Culicidae). Rev Bras Entomol.

[41] Smith GC, Eliason DA, Moore CG, Ihenacho EN (1988). Use of elevated temperatures to kill *Aedes albopictus* and *Aedes aegypti*. J Am Mosq Control Assoc.

[42] Woodhill AR (1948). Observations on the comparative survival of various stages of *Aëdes* (*Stegomyia*) *scutellaris* Walker and *Aëdes* (*Stegomyia*) *aegypti* Linnaeus at varying temperatures and humidities. Proc Linn Soc NSW.

[43] Otto M, Neumann RO (1905). Studien über gelbfieber in Brasilien. Z Hyg Infekt.

[44] Lambrechts L, Paaijmans KP, Fansiri T, Carrington LB, Kramer LD, Thomas MB, et al. Impact of daily temperature fluctuations on dengue virus transmission by *Aedes aegypti*. Proc Natl Acad Sci USA. 2011;108:7460–5. 10.1073/pnas.1101377108 PMC3088608 21502510

[45] Carrington LB, Seifert SN, Willits NH, Lambrechts L, Scott TW (2013). Large diurnal temperature fluctuations negatively influence *Aedes aegypti* (Diptera:Culicidae) life-history traits. J Med Entomol.

[46] Carrington LB, Seifert SN, Armijos MV, Lambrechts L, Scott TW (2013). Reduction of *Aedes aegypti* vector competence for dengue virus under large temperature fluctuations. Am J Trop Med Hyg..

[47] Couret J, Dotson E, Benedict MQ (2014). Temperature, larval diet, and density effects on development rate and survival of *Aedes aegypti* (Diptera: Culicidae). PLoS One.

[48] Braks MA, Honorio NA, Lourencqo-de-Oliveira R, Juliano SA, Lounibos LP. Convergent habitat segregation of *Aedes aegypti* and *Aedes albopictus* (Diptera: Culicidae) in southeastern Brazil and Florida. J Med Entomol. 2003;40:785–94. 10.1603/0022-2585-40.6.785 14765654

[49] Juliano SA, Lounibos LP, O'Meara GFA (2004). Field test for competitive effects of *Aedes albopictus* on *A. aegypti* in South Florida: differences between sites of coexistence and exclusion?. Oecologia.

[50] Chen CD, Nazni WA, Lee HL, Seleena B, Mohd Masri S, Chiang YF (2006). Mixed breeding of *Aedes aegypti* (L.) and *Aedes albopictus* Skuse in four dengue endemic areas in Kuala Lumpur and Selangor, Malaysia. Trop Biomed.

[51] Calderón-Arguedas O, Troyo A, Solano ME, Avendaño A, Beier JC (2009). Urban mosquito species (Diptera: Culicidae) of dengue endemic communities in the greater Puntarenas area, Costa Rica. Rev Biol Trop.

[52] Hwang JS, Hsu EL, Chen YR. Investigations on the density and breeding habitats of *Aedes* mosquitoes in dengue epidemic areas in Taiwan. Chin Aust J Public Health. 1995;14:228–36.

[53] Thavara U, Tawatsin A, Chansang C, Kong-ngamsuk W, Paosriwong S, Boon-Long J (2001). Larval occurrence, oviposition behavior and biting activity of potential mosquito vectors of dengue on Samui Island, Thailand. J Vector Ecol.

[54] Ponlawat A, Harrington LC (2005). Blood feeding patterns of *Aedes aegypti* and *Aedes albopictus* in Thailand. J Med Entomol.

[55] Scott TW, Takken W (2012). Feeding strategies of anthropophilic mosquitoes result in increased risk of pathogen transmission. Trends Parasitol.

[56] O'Meara GF, Evans LF, Gettman AD, Cuda JP (1995). Spread of *Aedes albopictus* and decline of *Ae. aegypti* (Diptera: Culicidae) in Florida. J Med Entomol.

[57] Daugherty MP, Alto BW, Juliano SA (2000). Invertebrate carcasses as a resource for competing *Aedes albopictus* and *Aedes aegypti* (Diptera: Culicidae). J Med Entomol.

[58] Bagny L, Delatte H, Quilici S, Fontenille D (2009). Progressive decrease in *Aedes aegypti* distribution in Reunion Island since the 1900s. J Med Entomol.

[59] Bagny L, Delatte H, Elissa N, Quilici S, Fontenille D (2009). *Aedes* (Diptera: Culicidae) vectors of arboviruses in Mayotte (Indian Ocean): distribution area and larval habitats. J Med Entomol.

[60] Raharimalala FN, Ravaomanarivo LH, Ravelonandro P, Rafarasoa LS, Zouache K, Tran-Van V (2012). Biogeography of the two major arbovirus mosquito vectors, *Aedes aegypti* and *Aedes albopictus* (Diptera, Culicidae), in Madagascar. Parasit Vectors.

[61] Lounibos LP (2002). Invasions by insect vectors of human disease. Annu Rev Entomol.

[62] Juliano SA, Lounibos LP (2005). Ecology of invasive mosquitoes: effects on resident species and on human health. Ecol Lett.

[63] Bargielowski IE, Lounibos LP, Carrasquilla MC. Evolution of resistance to satyrization through reproductive character displacement in populations of invasive dengue vectors. Proc Natl Acad Sci USA. 2013;110:2888–92. 10.1073/pnas.1219599110 PMC3581888 23359710

[64] Bargielowski IE, Lounibos LP, Shin D, Smartt CT, Carrasquilla MC, Henry A (2015). Widespread evidence for interspecific mating between *Aedes aegypti* and *Aedes albopictus* (Diptera: Culicidae) in nature. Infect Genet Evol.

[65] Roychoudhury S, Kobayashi M (2006). New findings on the developmental process of *Ascogregarina taiwanensis* and *Ascogregarina culicis* in *Aedes albopictus* and *Aedes aegypti*. J Am Mosq Control Assoc.

[66] Albicócco AP, Vezzani D (2009). Further study on *Ascogregarina culicis* in temperate Argentina: prevalence and intensity in *Aedes aegypti* larvae and pupae. J Invertebr Pathol.

[67] Dos Passos RA, Tadei WP (2008). Parasitism of *Ascogregarina taiwanensis* and *Ascogregarina culicis* (Apicomplexa: Lecudinidae) in larvae of *Aedes albopictus* and *Aedes aegypti* (Diptera: Culicidae) from Manaus, Amazon region, Brazil. J Invertebr Pathol.

[68] Teng HJ, Chung CL, Wang ST (1996). The distribution of dengue vectors and its possible explanation in the coastal area of Chiayi County. Chinese J Entomol.

[69] Ramos MM, Mohammed H, Zielinski-Gutierrez E, Hayden MH, Lopez JLR, Fournier M (2008). Epidemic dengue and dengue hemorrhagic fever at the Texas-Mexico border: results of a household-based seroepidemiologic survey, December 2005. Am J Trop Med Hyg.

[70] Aström C, Rocklöv J, Hales S, Béguin A, Louis V, Sauerborn R (2012). Potential distribution of dengue fever under scenarios of climate change and economic development. EcoHealth.

[71] Gubler DJ (2002). Epidemic dengue/dengue hemorrhagic fever as a public health, social and economic problem in the 21st century. Trends Microbiol.

[72] Gubler DJ (2002). The global emergence/resurgence of arboviral diseases as public health problems. Arch Med Res.

[73] Tsunoda T, Cuong TC, Dong TD, Yen NT, Le NH, et al. Winter refuge for *Aedes aegypti* and *Ae. albopictus* mosquitoes in Hanoi during winter. PLoS One. 2014;9:e95606. 10.1371/journal.pone.0095606 PMC3994068 24752230

[74] Central Weather Bureau (2009). 1897–2008 Statistics of Climate Changes in Taiwan.

